# AQP5 Is a Novel Prognostic Biomarker in Pancreatic Adenocarcinoma

**DOI:** 10.3389/fonc.2022.890193

**Published:** 2022-05-10

**Authors:** Guo Chen, Haiyang Song, Zelong Yang, Tianshu Du, Yu Zheng, Zifan Lu, Kunpeng Zhang, Di Wei

**Affiliations:** ^1^Department of Biopharmaceuticals, School of Pharmacy, Air Force Medical University, Xi’an, China; ^2^Department of Interventional Therapy, The Affiliated Yantai Yuhuangding Hospital of Qingdao University, Yantai, China; ^3^Department of Hepatobiliary Surgery, Xijing Hospital, Air Force Medical University, Xi’an, China; ^4^People’s Liberation Army (PLA) of Institute of Orthopedics Xijing Hospital, Air Force Medical University, Xi’an, China; ^5^Department of Urology, Xijing Hospital, Air Force Military Medical University, Xi’an, China; ^6^Medical Innovation Center, Fourth Military Medical Univeristy, Xi’an, China; ^7^Department of Catheterization Room, The Affiliated Yantai Yuhuangding Hospital of Qingdao University, Yantai, China

**Keywords:** AQP5, pancreatic adenocarcinoma, prognosis, tumor microenvironment, methylation

## Abstract

**Background:**

Pancreatic adenocarcinoma (PAAD) is a highly malignant tumor with a poor prognosis. The identification of effective molecular markers is of great significance for diagnosis and treatment. Aquaporins (AQPs) are a family of water channel proteins that exhibit several properties and play regulatory roles in human carcinogenesis. However, the association between Aquaporin-5 (AQP5) expression and prognosis and tumor-infiltrating lymphocytes in PAAD has not been reported.

**Methods:**

AQP5 mRNA expression, methylation, and protein expression data in PAAD were analyzed using GEPIA, UALCAN, HAP, METHSURV, and UCSC databases. AQP5 expression in PAAD patients and cell lines from our cohort was examined using immunohistochemistry and Western blotting. The LinkedOmics database was used to study signaling pathways related to AQP5 expression. TIMER and TISIDB were used to analyze correlations among AQP5, tumor-infiltrating immune cells, and immunomodulators. Survival was analyzed using TCGA and Kaplan–Meier Plotter databases.

**Results:**

In this study, we investigated AQP5 expression in PAAD and determined whether the expression of AQP5 is a strong prognostic biomarker for PAAD. We searched and analyzed public cancer databases (GEO, TCGA, HAP, UALCAN, GEPIA, etc.) to conclude that AQP5 expression levels were upregulated in PAAD. Kaplan–Meier curve analysis showed that high AQP5 expression positively correlated with poor prognosis. Using TIMER and TISIDB, we found that the expression of AQP5 was associated with different tumor-infiltrating immune cells, especially macrophages. We found that hypomethylation of the AQP5 promoter region was responsible for its high expression in PAAD.

**Conclusions:**

AQP5 can serve as a novel biomarker to predict prognosis and immune infiltration in PAAD.

## Introduction

Pancreatic adenocarcinoma (PAAD) is one of the most lethal malignancies and the fourth leading cause of cancer-related deaths in both men and women worldwide. Importantly, PAAD is projected to become the second leading cause of cancer-related deaths by 2030 (1, 2). Pancreatic ductal adenocarcinoma accounts for 95% of all pancreatic cancers (3). In addition, the 5-year survival rate of patients with pancreatic cancer is <5% (4). Despite substantial improvements in survival rates for other major cancer forms, PAAD survival rates have remained relatively unchanged since the 1960s. Pancreatic cancer is usually detected at an advanced stage, and most therapies are ineffective, contributing to poor overall prognosis (5). Thus far, these poor outcomes highlight the need to identify reliable early molecular markers to improve the prognosis of PAAD, which is vital.

**Figure 1:**
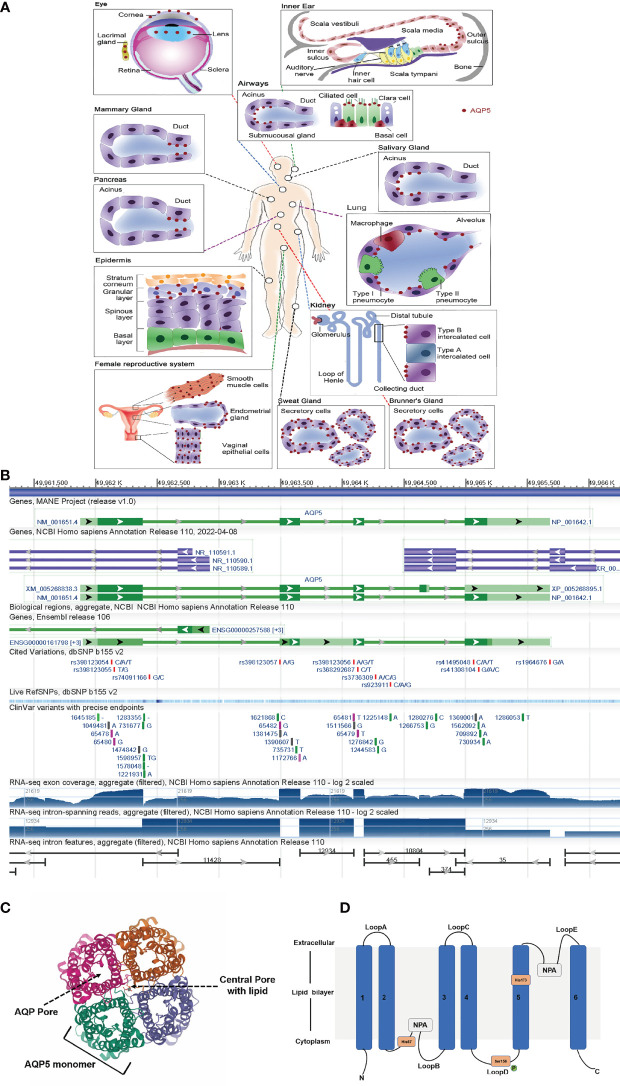
AQP5 is widely distributed among the human body. **(A)** AQP5 is widely distributed in the human body, such as in renal, digestive, and sensory organs. **(B)** Genomic regions, transcript, and products. **(C)** The AQP5 protein is composed of four monomers. **(D)** Topology map of the basic monomeric AQP5 fold.

Aquaporins (AQPs) are a family of integral membrane proteins that exhibit specific channel activities in water. They are highly selective transmembrane channels that transport water across cells and facilitate low-molecular-weight solutes (6). AQPs can play a significant part concerning metastasis, development, and angiogenesis of tumors (7, 8). As a member of AQPs, Aquaporin-5 (AQP5) is widely distributed in the human body, such as in renal, digestive, and sensory organs, as depicted in **Figure 1A** (9). AQP5 is located on human chromosome 12q3 and has a length of 1.8 kb, which has 4 exons and 3 introns (**Figure 1B**). The AQP5 protein is composed of four monomers that insert the cytomembrane as a tetramer (**Figure 1C**). Every monomer forms a central pore that interacts with two of its neighbors. Each monomer is composed of 265 amino acids, which are distributed along six transmembrane alpha-helices. The six transmembrane alpha-helices are connected by five loops (loops A–E). Loops B and E fold back into the membrane and form two half-helices, which contain two short hydrophobic stretches of amino acid residues, asparagine–proline–alanine (**Figure 1D**). AQP5 can form a special water channel that plays a vital role in the secretion of fluid from glands and maintains osmotic pressure inside and outside the cell (10).

In recent decades, AQP5 has been shown to be overexpressed in many cancers (lung, prostate, breast, etc.) (9, 11–13). Most studies have also reported that high AQP5 expression in digestive system tumors is frequently associated with increased disease progression, chemoresistance, and poor prognosis. For example, AQP5 is overexpressed in gastric cancer cell lines and tissue samples, especially in the intestinal histological type (14). Some studies have shown that AQP5 expression is upregulated in colon and colorectal cancer compared to normal colonic tissue, where its expression is rarely detected, improving drug resistance (15, 16). One study used AQP5 as a novel biomarker to predict poor clinical outcomes in colorectal cancer (17). A recent study suggested that AQP5 downregulation inhibits hepatocellular carcinoma metastasis and epithelial–mesenchymal transition (18). In addition, in PAAD samples, AQP5 was overexpressed in the apical membrane of intercalated cells and was related to tumor differentiation, suggesting that it may play a role in cancer progression (19, 20).

To the best of our knowledge, there have been no reports on the correlation of AQP5 with the prognosis of pancreatic cancer. Hence, to evaluate the potential of AQP5 as a novel diagnostic and prognostic marker of pancreatic cancer, we explored the whole genome expression data of patients with PAAD from Gene Expression Omnibus (GEO), The Cancer Genome Atlas (TCGA), UALCAN, and Gene Expression Profiling Interactive Analysis (GEPIA) databases. We found that AQP5 was significantly overexpressed in PAAD and that AQP5 expression was associated with tumor stage, tumor grade, and prognosis in patients with PAAD. The genes co-expressed with AQP5 were enriched in pathways involved in cell adhesion molecules, ECM–receptor interaction, the JAK-STAT signaling pathway, and Th17 cell differentiation. Furthermore, we used the TIMER web resource and the TISIDB database to analyze the correlation between AQP5 and tumor-infiltrated immune cells in the PAAD microenvironment. Our study indicates that AQP5 may be used as a novel prognostic biomarker and a new immune-associated therapeutic target for patients with PAAD.

## Materials and Methods

### The Cancer Genome Atlas

The TCGA database (https://portal.gdc.cancer.gov/) is a free website that provides a cancer genomics database, which contains the expression of genes, methylation, epigenetics, and proteome. The data on overall survival (OS), disease-specific survival (DSS), and progression-free interval (PFI) in pan-cancer were acquired *via* TCGA. The clinical data for PAAD were also obtained from TCGA.

### Gene Expression Profiling Interactive Analysis

The GEPIA database (https://gepia.cancer-pku.cn) is a free website that provides high-throughput RNA sequencing data for cancer. The expression of AQP5 in PAAD and normal pancreatic tissues was determined using GEPIA.

### UALCAN Analysis

UALCAN (http://ualcan.path.uab.edu/) is a free website for the online analysis of gene or protein expression and clinical data in tumors. In PAAD, the correlation between the expression of AQP5 and pathological T stage and histologic grade was analyzed. Correlation analysis of AQP5 mRNA expression with the promoter methylation status was performed. The correlation between AQP5 promoter methylation status, tumor grade, and tumor stage was also analyzed and compared.

### Human Protein Atlas

HPA is a free database that provides information on the expression of 24,000 proteins in tissues and organs. We used the HPA program to determine the distribution of AQP5 in PAAD and normal pancreatic tissues. We then acquired immunohistochemistry images of AQP5 proteins.

### UCSC Xena Analysis

UCSC Xena (https://xenabrowser.net/) is a free website for cancer genomics analysis that contains other databases, including TCGA, IGGC, and CCL. Visitors can analyze and download a database. We used this website to acquire AQP5 RNA-seq data in PAAD and normal pancreatic tissues from TCGA and GTEx for ROC curve analysis. The mutation, CAN, and methylation of AQP5 in PAAD were also analyzed.

### TIMER Analysis

The TIMER database (https://cistrome.shinyapps.io/timer/) was used to analyze the correlation between gene expression and immune infiltration in tumor tissues. We used this website to analyze the correlation between AQP5 expression and six tumor-infiltrating immune cells in PAAD. We analyzed the abundance of tumor-infiltrating immune cells with different somatic copy number aberrations of AQP5 in PAAD. Cox regression analysis of AQP5 and the six tumor-infiltrating immune cells was performed using this website. The expression correlation between AQP5 and markers of immune cell genes was also determined.

### TISIDB Analysis

The TISIDB database (http://cis.Hku.hk/TISIDB/) is a website that provides tumor immunity data. We used this website to analyze the relationship between AQP5 expression and 28 subtypes of tumor-infiltrating immune cells in different types of tumors. We also analyzed the correlation between AQP5 expression or methylation and tumor immune infiltration in PAAD.

### METHSURV Analysis

The MethSurv database is a website for investigating the correlation between methylation biomarkers and the prognosis of patients with cancer. We used it to analyze DNA methylation at the CpG sites of AQP5 in PAAD and its prognostic value.

### Kaplan–Meier Plotter Database Analysis

The Kaplan–Meier Plotter database (http://kmplot.com/analysis/) is a website that provides survival analysis of 54,675 genes in 21 tumor types. We performed a survival analysis of AQP5 expression levels in enriched immune cells in PAAD.

### LinkedOmics Database Analysis

The LinkedOmics database (http://www.linkedomics.org/login.php) is a website that analyzes TCGA data. We searched for genes related to AQP5 expression in PAAD. The differentially expressed genes related to AQP5 were analyzed using Gene Ontology (GO) and classified as molecular function (MF), cellular component (CC), and biological process (BP). Gene set enrichment analysis and Kyoto Encyclopedia of Genes and Genomes pathway enrichment analyses were performed using the link interpreter module.

### Cell Culture, Western Blot, Immunohistochemistry, Immunofluorescence, and RT-PCR

The PAAD cell line (PANC1) and human pancreatic ductal epithelial cell line (HPDE6) were obtained from the ATCC. All the cell lines were cultured in DMEM containing 1% penicillin–streptomycin and 10% FBS. The normal pancreatic tissues of PAAD patients were obtained from Xijing Hospital. Western blotting, RT-PCR, immunofluorescence, and immunohistochemistry were performed as previously reported (21–23). The following antibodies were used: AQP5 (Abcam, Ab92320) and tubulin (Proteintech, 10094-1-AP). AQP5 primer sequence, Forward 5’-3’: GCCACCTTGTCGGAATCTACT; Reverse 5’-3’: GGCTCATACGT-GCCTTTGATG.

### AQP5 Knockdown, Transwell Migration, and Invasion

AQP5 knockdown and Transwell assays were performed as previously reported (23, 24). Small interfering RNA (si-RNA) against AQP5 and corresponding negative control (si-NC) were purchased from Tisingke Biotechnology Co., Ltd. (Beijing, China). si-AQP5 sequences and si-NC sequences were transfected into cells using Lipofectamine 3000 Reagent. si-AQP5-1 primer sequence, Forward 5’-3’: GGCACGUAUGAGCCUGACGTT; Reverse5’-3’: CGUCAGGCUCAUACGUGCCTT. si-AQP5-2 primer sequence, Forward 5’-3’: GCAUCUUCGCCUCCACUGATT; Reverse5’-3’: UCAGUGGAGG-CGAAGAUGCTT.

### Statistical Analysis

The expression of AQP5 in pan-cancer and normal tissues was acquired *via* the UCSC Nena website; the R package was used to analyze the difference. The ROC curve was plotted using R software. The survival data and clinical data were obtained *via* TCGA, the R package was used to analyze correlation, and the *p*-value was calculated by a log-rank test. Logistic regression analysis was used to analyze the relationship between AQP5 levels and clinical characteristics. Univariate and multivariate Cox regression analyses were performed to confirm independent risk factors. The measurement data are presented as the mean ± SD. Spearman’s correlation analysis was used for nonparametric tests. Statistical significance was set at *p* < 0.05.

## Results

### The AQP5 mRNA Expression in Different Cancers

**Figure 2:**
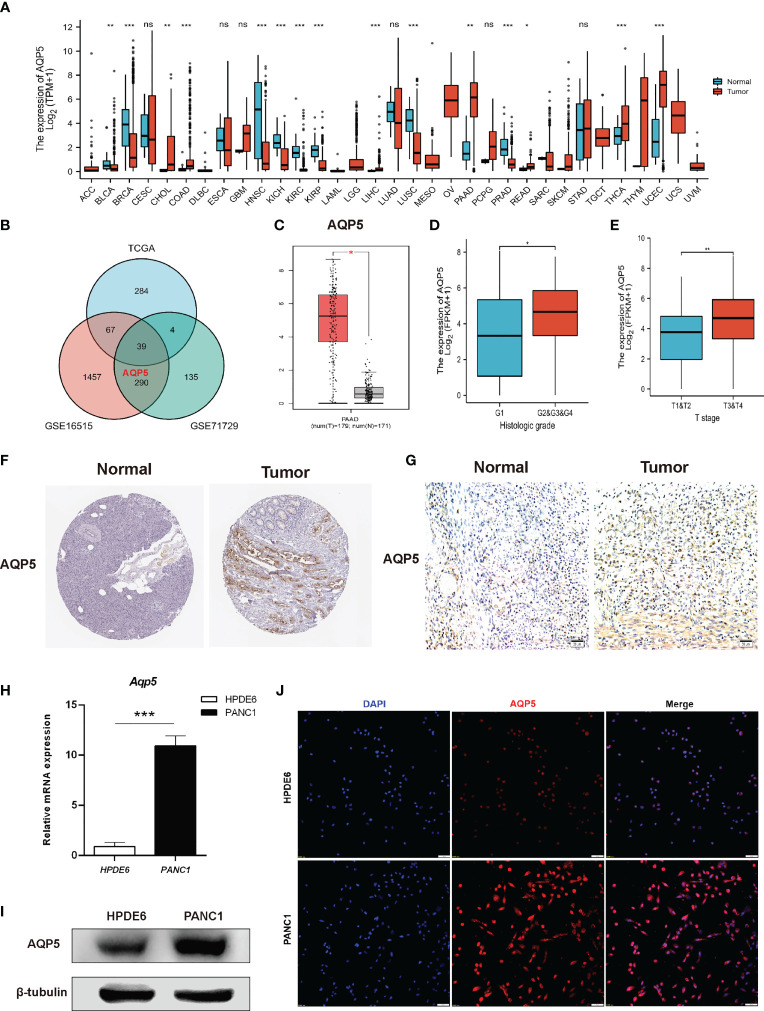
The expression of gene AQP5 mRNA in different kinds of tumors and its relationship with clinical parameters in PAAD. **(A)** Human expression levels of AQP5 in 33 various malignant tumor types from The Cancer Genome Atlas (TCGA) database were analyzed by the Tumor Immune Estimation Resource (TIMER). **(B)** Venn diagram showing the common differential expression genes in three public databases (TCGA, GES16515, and GES71729). **(C)** The expression of AQP5 in PAAD and normal pancreatic tissues, respectively. **(D, E)** Association between AQP5 expression and clinicopathologic characteristics with PAAD patients’ cancer grade **(D)** and pathological T stage **(E)**. The immunohistochemistry of AQP5 in PAAD and normal pancreatic tissue **(F, G)**. **(H–J)** AQP5 mRNA and protein levels were overexpressed compared with real-time PCR **(H)**, Western blotting **(I)**, and immunofluorescence **(J)**. ^*^p < 0.05, ^**^p < 0.01, ^***^p < 0.001, NS, not significant.

We used the UCSC Xena database to analyze AQP5 mRNA expression in pan-cancer and normal tissues. The results indicated that AQP5 mRNA expression was very high in most tumors, such as adrenocortical carcinoma, pancreatic carcinoma, cholangiocarcinoma, and colon adenocarcinoma, compared to the corresponding normal tissues (**Figure 2A**). In TCGA, GSE16515, and GES71729 datasets, we found that AQP5 was expressed at higher levels in PAAD tissues than in normal tissues (**Figure 2B**). We also found that AQP5 expression in PAAD was higher than that in normal pancreatic tissues in the TIMER database (**Figure 2C**). The AQP5 expression was higher in histologic G2, G3, and G4 patients than that in G1 patients (**Figure 2D**). AQP5 expression was higher in patients with pathological T3 and T4 stage disease than in those with T1 and T2 stage disease (**Figure 2E**). In the HPA database, we found that AQP5 protein expression levels were significantly increased in PAAD tissues compared to normal pancreatic tissues (**Figure 2F**). The protein levels of AQP5 were examined in pancreatic cell lines and tissues. **Figure 2G** shows that AQP5 protein levels were overexpressed compared to immunohistochemistry in clinical PAAD tissue. Similarly, we confirmed this in the PAAD cell lines. AQP5 mRNA and protein levels were overexpressed compared with real-time PCR, Western blotting, and immunofluorescence (**Figures 2H–J**).

### The Correlation Between the AQP5 Expression and Survival

**Figure 3:**
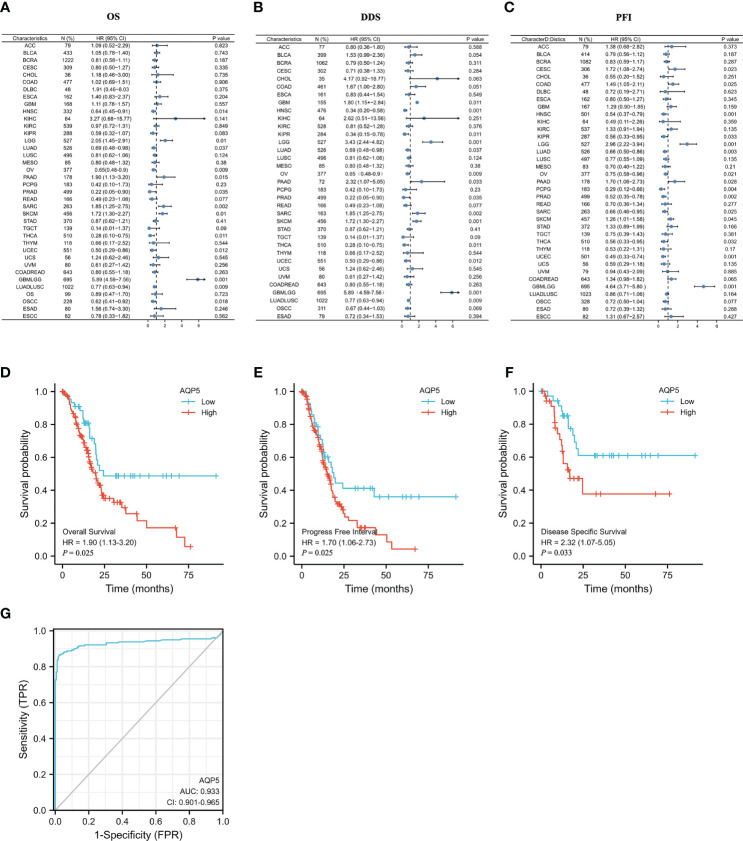
The prognostic value of AQP5 expression in patients with PAAD. **(A–C)** Forest plot of the prognostic values in different tumor types of AQP5. **(D–F)** Kaplan–Meier survival analysis revealed that low AQP5 expression in patients with PAAD had a longer overall survival **(D)**, progression-free interval **(E)**, and disease-specific survival **(F)**. **(G)** The ROC curve of the prognostic values in patients with PAAD of AQP5.

Kaplan–Meier analysis was used to compare the different prognoses between the AQP5 high- and low-expression groups in different types of tumors. A forest plot showed AQP5 as a risk factor for OS, PFI, and DSS prognostic values in PAAD (**Figures 3A–C**). We plotted survival curves for OS (HR = 1.9, *p* = 0.015), DSS (HR = 2.32, *p* = 0.033), and PFI (HR = 1.7, *p* = 0.028) (**Figures 3D–F**). The resulting ROC curve is shown in **Figure 3G**. These results showed that AQP5 may predict a worse prognosis in patients with PAAD.

### Correlation Between AQP5 Expression and Clinical Index in PAAD

We analyzed the association between AQP5 expression and the PAAD clinical index using logistic regression. The results indicated that high expression AQP5 was significantly associated with pathological T stage classification (OR = 0.133, *p* = 0.036 for T2 vs. T1; OR = 3.455, *p* = 0.013 for T3 vs. T2) (**Table 1**).

**Table 1:** The logistic analysis of correlation between AQP5 and clinical characteristics in patients with PAAD.

Characteristics	Total (*N*)	Odds Ratio (OR)	*p*-value
T stage (T2 vs. T1)	31	0.133 (0.016–0.790)	0.036^*^
T stage (T3 vs. T2)	166	3.455 (1.360–9.993)	0.013^*^
N stage (N1 vs. N0)	173	0.811 (0.417–1.567)	0.534
M stage (M1 vs. M0)	84	5.294 (0.742–106.100)	0.144
Gender (Male vs. Female)	178	1.314 (0.727–2.382)	0.366
Age (>65 vs. ≤65)	178	1.645 (0.912–2.991)	0.100
Pathologic stage (Stage II vs. Stage I)	167	1.717 (0.682–4.568)	0.259
Histologic grade (G2 vs. G1)	126	2.199 (0.964–5.231)	0.066

*p < 0.05.

### Univariate and Multivariate Cox Regression Analysis

We investigated the prognostic risk factors for PAAD using the Cox regression analysis. Univariate Cox regression analysis indicated that pathologic stage II (HR = 2.333, *p* = 0.033), histologic G3 (HR = 2.625, *p* = 0.007), and AQP5 (HR = 1.142, *p* = 0.009) were factors that affected the survival of patients with PAAD. Multivariate Cox regression analysis showed that histologic G3 (HR = 2.191, *p* = 0.007) and AQP5 (HR = 1.134, *p* = 0.009) were independent risk factors influencing the survival of patients with PAAD (**Table 2**).

**Table 2:** Univariate and multivariate Cox regression analysis of AQP5 expression for overall survival in patients with PAAD.

Characteristics	Total (*N*)	HR (95% CI) Univariate analysis	*p*-value Univariate analysis	HR (95% CI) Multivariate analysis	*p*-value Multivariate analysis
Gender	178				
Female	80	Reference			
Male	98	0.809 (0.537–1.219)	0.311		
Age	178				
≤65	93	Reference			
>65	85	1.290 (0.854–1.948)	0.227		
T stage	176				
T1	7	Reference			
T2	24	1.451 (0.311–6.779)	0.636		
T3	142	2.742 (0.667–11.269)	0.162		
T4	3	1.462 (0.131–16.289)	0.757		
Pathologic stage	175				
Stage I	21	Reference			
Stage II	146	2.333 (1.069–5.089)	0.033^*^		
Stage III	3	1.255 (0.153–10.275)	0.832		
Stage IV	5	1.566 (0.321–7.637)	0.579		
Histologic grade	176				
G1	31	Reference			
G2	95	1.959 (1.007–3.808)	0.048	1.644 (0.835–3.236)	0.150
G3	48	2.625 (1.304–5.283)	0.007^*^	2.191 (1.076–4.463)	0.031*
G4	2	1.651 (0.211–12.893)	0.632	1.715 (0.220–13.397)	0.607
AQP5	178	1.142 (1.033–1.262)	0.009^*^	1.134 (1.014–1.268)	0.027*
Race	174				
Asian Black or African American	17	Reference			
White	157	1.161 (0.582–2.318)	0.672		

*p < 0.05.

### AQP5 Expression Was Correlated With Immune Cell Infiltrations in PAAD

**Figure 4:**
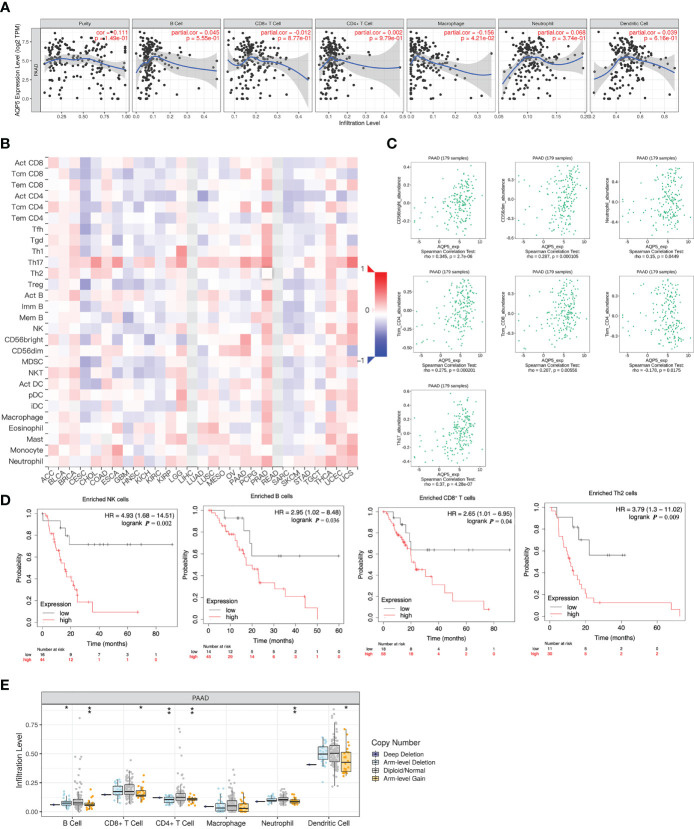
Correlation between AQP5 expression and immune cell infiltrations in PAAD. **(A)** The expression of AQP5 in PAAD tissues positively correlated with tumor immune infiltration levels of macrophage. **(B)** Relationship between AQP5 expression and 28 subtypes of tumor-infiltrating immune cells in different kinds of tumors. **(C)**. The CD56 (bright) cell, CD56 (dim) cell, neutrophil, CD4^+^ central memory T cell, CD8^+^ central memory T cell, CD4^+^ effective memory T cell, and T helper cell 17 had significant Spearman’s correlation with AQP5 expression in PAAD. **(D)** Low AQP5 expression levels in PAAD in enriched B cell, natural killer cell, CD8^+^ T cell, and Th2 cell cohorts had a favorable prognosis. **(E)** The abundance of tumor-infiltrating immune cells with different somatic copy number aberrations of AQP5 in PAAD, *p < 0.05, **p < 0.01.

We used the TIMER database to analyze the relationship between AQP5 expression and immune cell infiltration in various cancers. The results indicated that the AQP5 expression was significantly correlated with gene markers of macrophage (*r* = 0.068, *p* = 4.32e-0.2) (**Figure 4A**). Using the TISIDB database, we analyzed the correlation between AQP5 expression and 28 subtypes of tumor-infiltrating immune cells in different types of tumors (**Figure 4B**). The results showed that CD56 (bright) cells (*r* = 0.345, *p* = 2.7e-6), CD56 (dim) cells (*r* = 0.287, *p* < 0.01), neutrophils (*r* = 0.15, *p* = 0.045), CD4^+^ central memory T cells (*r* = 0.275, *p* < 0.01), CD8^+^ central memory T cells (*r* = 0.207, *p* = 0.006), CD4^+^ effective memory T cells (*r* = −0.178, *p* = 0.018), and T helper cell 17 (*r* = 0.37, *p* = 4.28e-7) were significantly correlated with AQP5 expression in PAAD (**Figure 4C**). We used the Kaplan–Meier plotter database to investigate the survival of AQP5 expression, followed by subgroups of immune cells in PAAD. The results showed that low AQP5 expression levels in PAAD-enriched B cells, natural killer cells, CD8^+^ T cells, and Th2 cell cohorts were associated with a favorable prognosis (**Figure 4D**). We also found that the abundance of tumor-infiltrating immune cells was affected by different somatic copy number aberrations of AQP5 in PAAD (**Figure 4E**). In addition, we used the TIMER database to investigate the relationship between AQP5 expression and the gene markers of tumor-infiltrating immune cells in PAAD (**Table 3**). We found that AQP5 expression correlated with the gene markers of B cells (CD79A), TAM (CD68), Th1 (STAT1), and neutrophils (CEACAM8) in PAAD.

**Table 3:** The correlation analysis between AQP5 expression and immune cell-associated gene markers in PAAD.

		None		Purity	
		cor	*p*	cor	*p*
B cell	CD19	−0.077	0.304	−0.170	0.026
	CD79A	−0.072	0.039^*^	−0.196	0.010^*^
CD8^+^ T cell	CD8A	−0.028	0.709	−0.204	0.007
	CD8B	−0.020	0.795	−0.194	0.011
Dendritic cell	ITGAX	0.090	0.233	−0.279	0.000
	NRP1	−0.042	0.577	−0.224	0.001
	CD1C	0.030	0.686	−0.197	0.009
	HLA-DPA1	−0.012	0.878	−0.263	0.000
	HLA-DRA	0.031	0.682	0.272	0.003
	HLA-DQB1	0.112	0.134	−0.223	0.003
	HLA-DPB1	0.045	0.551	−0.050	0.000
M1 Macrophage	PTGS2	0.135	0.072	0.114	0.138
	IRF5	0.055	0.464	−0.038	0.624
	NOS2	0.143	0.056	0.004	0.959
M2 Macrophage	MS4A4A	0.027	0.724	−0.344	0.000
	VSIG4	0.041	0.586	−0.376	0.000
	CD163	−0.033	0.664	−0.318	0.000
Monocyte	CSF1R	−0.047	0.530	−0.320	0.000
	CD86	0.021	0.776	−0.302	0.000
Natural killer cell	KIR2DS4	−0.166	0.026	−0.029	0.703
	KIR3DL3	0.069	0.356	−0.149	0.052
	KIR3DL2	0.046	0.537	−0.162	0.034
	KIR3DL1	−0.180	0.016	−0.105	0.171
	KIR2DL4	0.076	0.313	0.023	0.764
	KIR2DL3	0.011	0.880	0.101	0.189
	KIR2DL1	0.040	0.597	−0.061	0.427
Neutrophils	CCR7	−0.097	0.197	−0.170	0.026
	ITGAM	0.046	0.537	−0.375	0.000
	CEACAM8	0.230	0.002^*^	−0.196	0.009^*^
T cell (general)	CD3D	0.054	0.476	−0.203	0.007
	CD3E	−0.006	0.940	−0.218	0.004
	CD2	−0.011	0.884	−0.251	0.001
T cell exhaustion	CTLA4	0.022	0.760	−0.186	0.014
	LAG3	−0.061	0.410	−0.077	0.313
	HAVCR2	0.010	0.890	−0.339	0.000
	GZMB	0.035	0.639	−2.060	0.007
	PDCD1	0.048	0.522	−0.185	0.015
TAM	CCL2	0.033	0.656	−0.148	0.052
	IL10	0.108	0.149	−0.159	0.037
	CD68	0.159	0.034^*^	−0.321	0.000^*^
Tfh	BCL6	0.168	0.024	−0.008	0.910
	IL21	−0.022	0.775	−0.124	0.106
Th1	TBX21	−0.063	0.402	−0.172	0.024
	STAT4	−0.232	0.002	−0.136	0.076
	STAT1	0.200	0.007^*^	−0.221	0.004^*^
	IFNG	0.048	0.510	−0.236	0.002
	IL13	−0.099	0.186	0.018	0.811
Th2	GATA3	0.121	0.106	−0.097	0.204
	STAT6	0.384	0.000	0.009	0.901
	STAT5A	0.119	0.111	−0.176	0.021
Th17	STAT3	−0.041	0.590	−0.135	0.076
	IL17A	0.027	0.725	−0.075	0.326
Treg	FOXP3	0.000	0.998	−0.246	0.002
	CCR8	0.019	0.797	−0.207	0.006
	STAT5B	−0.139	0.063	−0.028	0.715
	TGFB1	0.061	0.418	−0.017	0.827

*p < 0.05.

### Mutation, Copy Number Variation, and Methylation Analysis of AQP5

**Figure 5:**
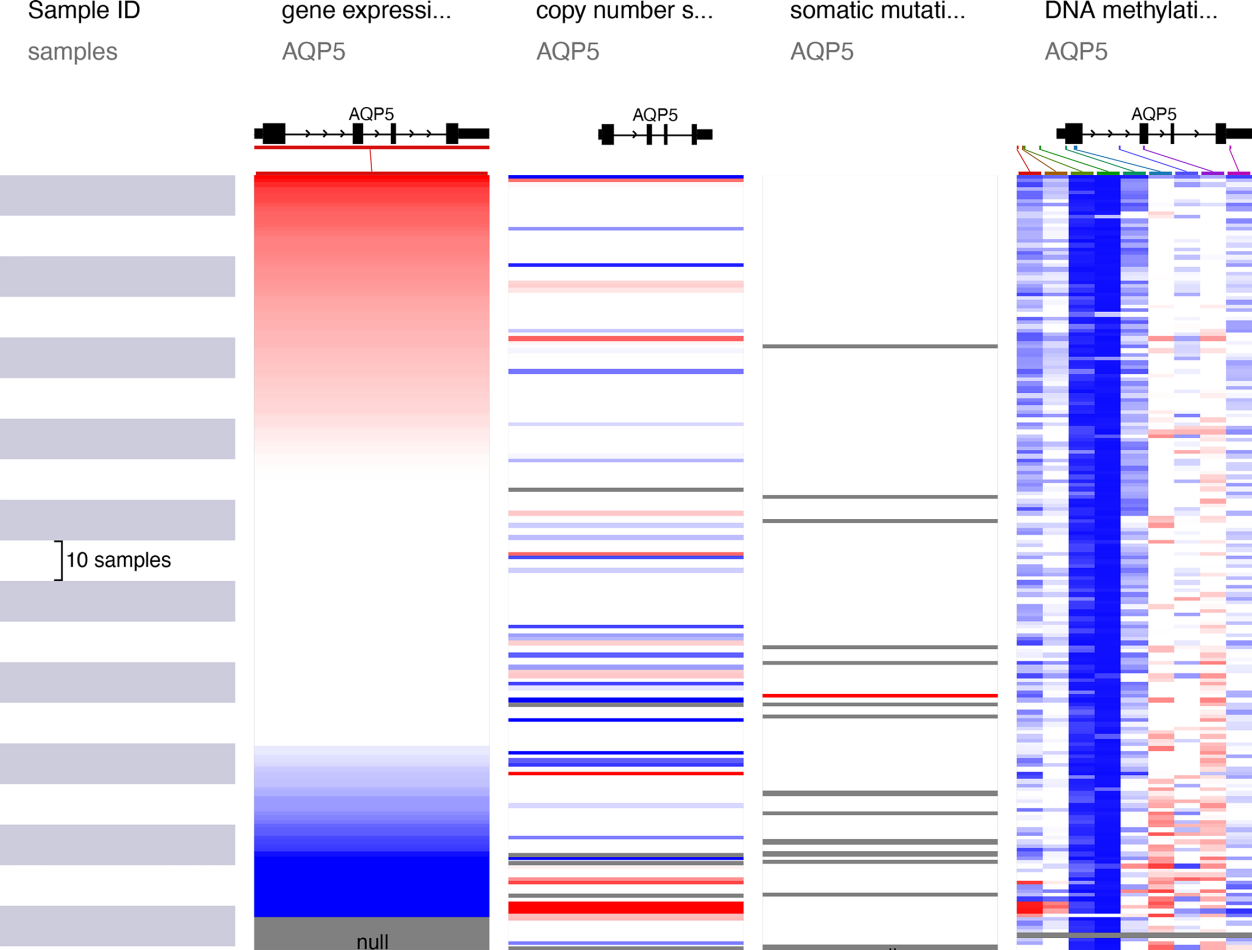
The heatmap demonstrated the mutation, CNV, and methylation analysis of AQP5 in PAAD.

AQP5 expression is significantly elevated in PAAD. We assessed the cause of the elevated PAAD levels. Gene mutations, copy number variation (CNV), and DNA methylation are critically involved in genetic and epigenetic regulation and are highly associated with cancer progression. We verified DNA methylation, gene mutation, and CNV levels of the AQP5 in PAAD using the UCSC Xena database. The heatmap indicated that the expression of AQP5 mRNA was correlated with DNA methylation but not with CNV and somatic mutations in PAAD (**Figure 5**). Therefore, we suggest that DNA methylation may contribute to elevated levels of AQP5 in PAAD.

### AQP5 Methylation and Its Prognostic Value in PAAD

**Figure 6:**
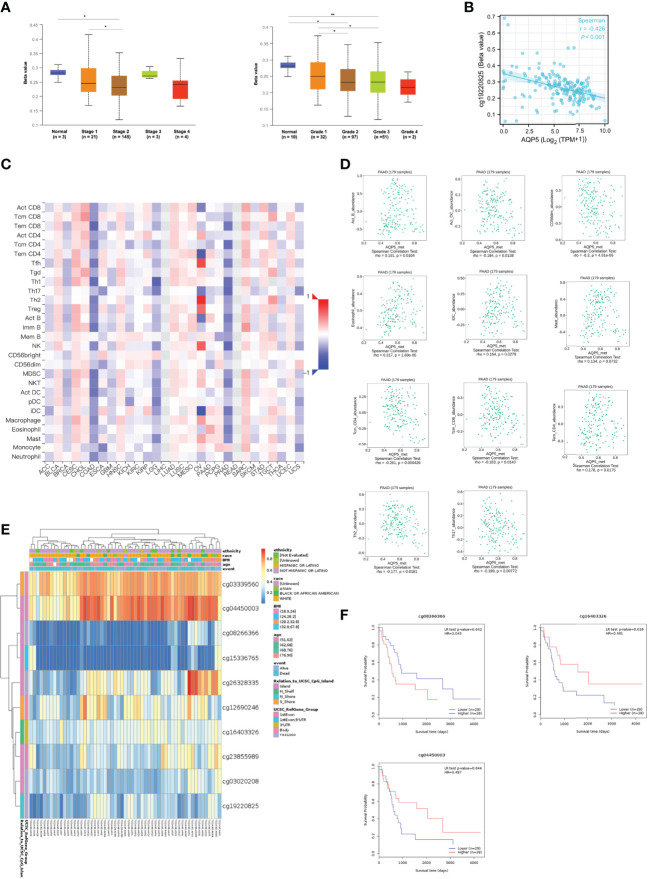
DNA methylation levels of AQP5 and its prognostic value in PAAD. The difference of promoter methylation level of AQP5 between tumor stage **(A)** and grade **(B)** in PAAD. **(C)** The expression of AQP5 correlated with AQP5 promoter methylation. **(D)** The heatmap of DNA methylation at CpG sites of AQP5 in PAAD. **(E)** The survival analysis of AQP5 promoter methylation in patients with PAAD. **(F)** Relationship between AQP5 promoter methylation and 28 subtypes of tumor-infiltrating immune cells in different kinds of tumors. **(G)** Association between the methylation status of AQP5 with immune infiltrates in PAAD. *p < 0.05, **p < 0.01.

The methylation status of AQP5 was low in high-stage and high-grade tumors (**Figure 6A**). Furthermore, correlation analysis indicated that AQP5 mRNA expression was significantly negatively correlated with its methylation status (**Figure 6B**). We then used the TISIDB database to further explore the relationship between AQP5 levels and 28 tumor-infiltrating immune cell subtypes (**Figure 6C**). These results showed that AQP5 is associated with some immune cell subtypes in PAAD (**Figure 6D**). The results revealed that Th2 cells (rho = −0.177, *p* = 0.0181), Th17 cells (rho = −0.199, *p* = 0.00772), Tem-CD4 cells (rho = 0.178, *p* = 0.0175), Tcm-CD4 cells (rho = −0.199, *p* = 0.000426), Tcm-CD8 cells (rho = −0.261, *p* = 0.0143), iDC (rho = 0.164, *p* = 0.0279), eosinophils (rho = 0.317, *p* = 1.69e-05), CD56 cells (rho = −0.3, *p* = 4.91e-05), Act-DCs (rho = −0.184, *p* = 0.0138), Act-B cells (rho = 0.191, *p* = 0.0104), and AQP5 were moderately correlated. The heatmap shows the methylation of CpG islands in AQP5 (**Figure 6E**). Consistently, CpG sites of AQP5, including cg04450003 and cg16403326, indicated that PAAD methylation at these CpG sites was correlated with favorable prognosis in patients with PAAD. However, cg08266366 indicated that PAAD methylation at these CpG sites correlated with poor prognosis (**Figure 6F** and **Table 4**). These results reveal that the methylation levels of AQP5 act as an effective prognostic biomarker for patients with PAAD, demonstrating that AQP5 may play a pivotal role in tumor progression.

**Table 4:** The prognostic value of CpG in AQP5.

Name	HR	CI	*p*-value	LR_test_*p*-value	Best_split	UCSC_RefGene_Group	Relation_to_UCSC_CpG_Island
cg03020208	2.187	(0.888; 5.387)	0.089	0.067	q25	TSS1500	Island
cg03339560	0.528	(0.252; 1.104)	0.09	0.1	q25	Body	S_Shore
cg04450003	0.497	(0.25; 0.991)	0.047*	0.044	Median	1stExon	Island
cg08266366	2.043	(1.02; 4.09)	0.044*	0.042	Median	TSS1500	Island
cg12690246	1.727	(0.875; 3.41)	0.12	0.11	Mean	Body	S_Shore
cg15336765	1.314	(0.608; 2.838)	0.49	0.48	q25	1stExon;5’UTR	Island
cg16403326	0.461	(0.229; 0.929)	0.03*	0.026	Median	3’UTR	N_Shelf
cg19220825	1.26	(0.567; 2.802)	0.57	0.56	q25	TSS1500	N_Shore
cg23855989	0.888	(0.437; 1.804)	0.74	0.74	q25	1stExon	Island
cg26328335	1.704	(0.772; 3.763)	0.19	0.17	q25	TSS1500	Island

*p < 0.05.

### Enrichment Analyses of Genes Co-Expressed With AQP5 in PAAD

**Figure 7:**
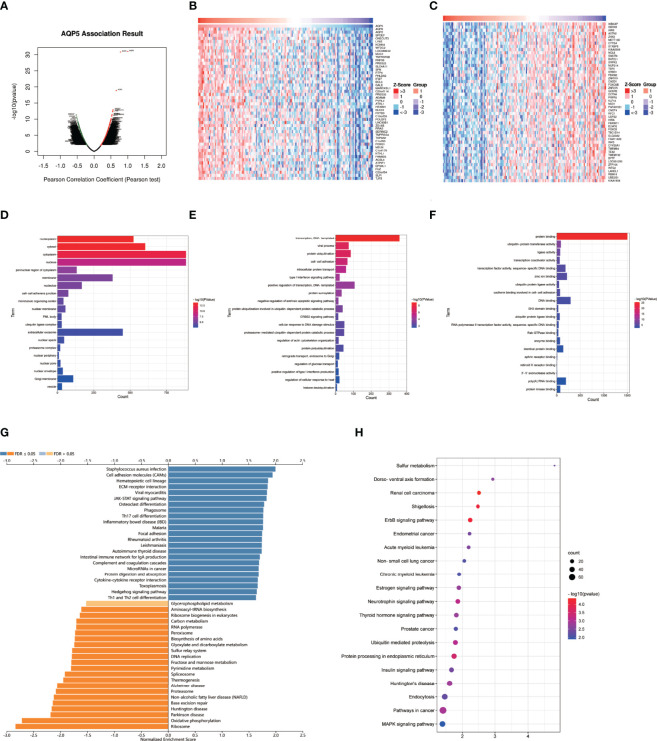
AQP5 co-expressed gene and functional enrichment analysis in PAAD. **(A)** The volcano map of AQP5 gene co-expression. **(B, C)** The heatmap of the top 50 positive and negative AQP5 gene co-expression. **(D–F)** The GO analysis of AQP5 gene co-expression in molecular function (MF), cellular component (CC), and biological process (BP). **(G)** AQP5 co-expression genes were annotated by Gene Ontology (GO) analysis. **(H)** The top 20 KEGG pathway analysis of AQP5 gene co-expression.

**Figure 8:**
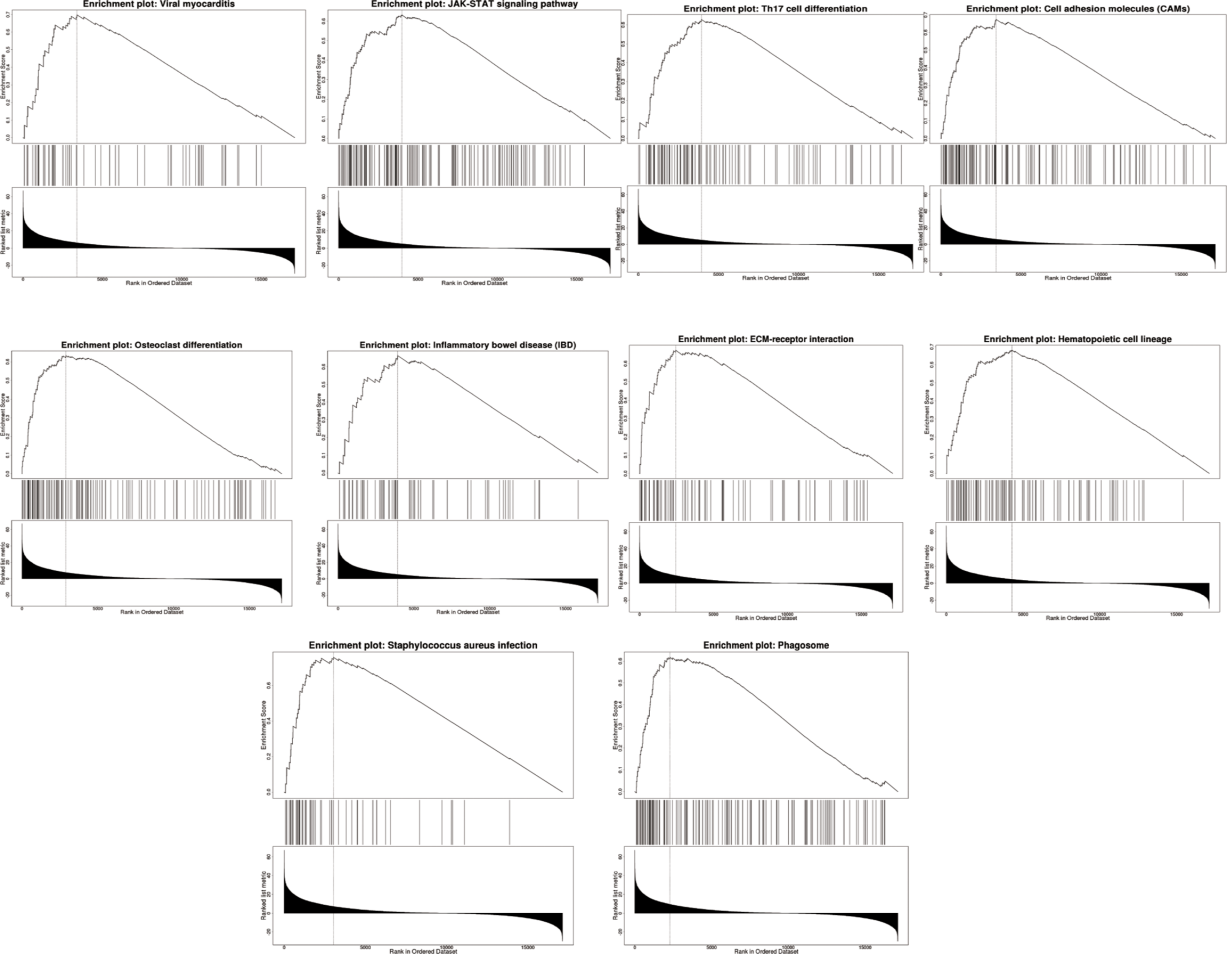
Top 10 positively correlated GSEA enrichment plots.

We used LinkedOmics to perform enrichment analysis of co-expression genes associated with AQP5 in PAAD. As shown in the volcano plot (**Figure 7A**), 1,424 positively associated genes (red) and 1,592 negatively associated genes (green) were identified in PAAD (*p* < 0.05, FDR < 0.01). A heatmap was used to display the top 50 positively and negatively correlated genes in PAAD (**Figures 7B, C**). GO-BP analysis showed that co-expressed genes were involved in transcription, DNA-templated viral process protein ubiquitination, cell–cell adhesion, intracellular protein transport, and the type I interferon signaling pathway. GO-CC analysis showed that these genes were mainly enriched in the nucleoplasm, cytosol, cytoplasm, nucleus, and perinuclear regions of the cytoplasm and membrane. GO-MF analysis showed that these genes were predominantly enriched for protein binding, ubiquitin-protein transferase activity, ligase activity, transcription coactivator activity, transcription factor activity, sequence-specific DNA binding, and zinc ion binding (**Figures 7D–F**). GO term annotation showed that co-expressed genes of AQP5 were mainly involved in *Staphylococcus aureus* infection, cell adhesion molecules, hematopoietic cell lineage, ECM–receptor interaction, viral myocarditis, JAK-STAT signaling pathway, osteoclast differentiation, phagosome, Th17 cell differentiation, and inflammatory bowel disease (**Figure 7G**). KEGG pathway analysis showed that the co-expressed genes were enriched in renal cell carcinoma, ErbB signaling pathway, Shigellosis protein processing in the endoplasmic reticulum, neurotrophin signaling pathway, and ubiquitin-mediated proteolysis pathways in cancer in PAAD (**Figure 7H**). **Figure 8** shows the detailed GSEA enrichment plots.

### AQP5 Knockdown Inhibits Migration and Invasion of PAAD Cells *In Vitro*

**Figure 9:**
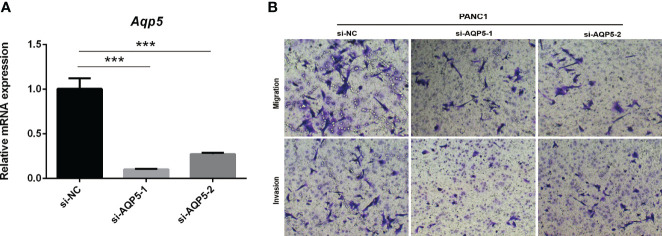
AQP5 knockdown inhibits migration and invasion of PAAD cells *in vitro*. Knockdown of AQP5 significantly impaired the migration and invasion capabilities of PANC-1 cells **(A, B)**. ***p < 0.001.

To investigate whether AQP5 affects the migration and invasion of PAAD cells, we performed Transwell assays. As shown in **Figures 9A, B**, AQP5 knockdown significantly impaired the migration and invasion capabilities of PANC-1 cells. These results suggest that AQP5 promotes migration and invasion of PAAD cells.

## Discussion

PAAD is a common malignant tumor of the digestive system and one of the deadliest diseases in the world (25). Its clinical symptoms mainly depend on the site of cancer, including abdominal fullness, discomfort, and pain. Owing to its insidious onset, rapid progression, and high malignancy, only 9.7% of patients are diagnosed early (26). The 5-year overall survival rate increased from 3% in the 1970s to 9% in 2020 (27). The 5-year survival rate for patients with advanced disease is still 3% (27). The incidence of PAAD is likely to increase in the future owing to lifestyle changes, such as alcohol consumption, high-fat diets, and high-glucose diets. However, 80% of patients are diagnosed with metastasis, and no more than 20% of patients can be treated surgically (28). Therefore, early diagnosis and treatment are key to improving the prognosis of patients with PAAD. With the development of genomics, tumor-related genes are constantly being explored. It is of great significance to identify reliable biological markers for early diagnosis and prognosis of PAAD.

The AQP family is a highly conserved membrane protein that was discovered in red blood cells in the 1950s (29). AQPs are primarily involved in the bidirectional movement of water and small solutes on the cell membrane, which are found in epithelial, endothelial, and other cell types in mammals, maintaining various biological functions (29). Previous studies have reported that skin aging is accompanied by reduced AQP5 expression and that the presence of AQP5 can promote the proliferation and differentiation of skin cells (30). On the other hand, since the occurrence of malignant tumors is related to the microenvironment, in recent years, there have been increasing reports of AQP5 in relation to the occurrence of tumors. In small-cell lung cancer, silencing AQP5 can inhibit the invasion and migration of tumor cells, which also inhibits angiogenesis and cell proliferation signaling pathways (31). In prostate cancer, AQP5 expression has been correlated with tumor grade, circulating tumor cells, lymph node metastasis, Gleason score, and PSA (32). However, the use of AQP5 as a marker for the early diagnosis and prognosis of PAAD has not been reported.

In this study, 39 upregulated genes, including AQP5, were identified using the TCGA and GES databases using a Venn diagram. We analyzed the correlation between AQP5 and PAAD clinical data from TCGA, GEPIA, UALCAN, and HPA databases. The results showed that AQP5 mRNA and protein levels were highly expressed in PAAD patients and were verified in the PAAD patient tissues and cell lines in our center. In addition, we found that higher AQP5 expression was associated with higher pathological T-stage and clinical stage, which also had unfavorable survival. These results suggest that AQP5 can be used as a prognostic marker for patients with PAAD.

Using TIMER and TISIDB, we analyzed the relationship between AQP5 and infiltrating immune cells in PAAD. We found that the expression of AQP5 was associated with macrophages, whereas analysis of immune-related markers suggested that the expression of AQP5 was associated with activation markers of TAM cells, B cells, Th1 cells, and neutrophils. We also found that AQP5 expression was positively correlated with different immune cell subtypes, such as CD56^dim^ NK cells, CD56^bright^ NK cells, CD4+ Tcm, CD8+ Tcm, and Th17 cells. CD4+ Tem expression was negatively correlated with AQP5 expression. CD56 bright NK cells can differentiate into CD56dim NK cells, which play a cytotoxic role (33). Therefore, NK cells are involved in inflammation and innate immunity. In contrast, the activation of T1, neutrophils, and B-cell-related molecular markers reflects the role of AQP5 in promoting inflammation. Th17 cells secrete IL-17A, which stimulates vascular endothelial growth factor (VEGF), resulting in tumor angiogenesis (34). In PAAD, one study reported that IL-22 secreted by Th17 cells was correlated with unfavorable survival (34). Moreover, TAM can promote tumor proliferation, angiogenesis, and metastasis (35). Recent research has demonstrated that an inflammatory environment can promote the occurrence and development of tumors (21). Therefore, we speculated that AQP5 expression promoted the generation and accelerated PAAD progression. Simultaneously, the activation of T1, neutrophils, and B-cell-related molecular markers reflects the role of AQP5 in promoting the inflammatory environment, which can promote the occurrence and development of tumors. Although CD4^+^ and CD8^+^ Tcm can inhibit tumor progression (36), we also found that AQP5 expression might reduce CD4^+^ Tem in our study. These results indicate that AQP5 plays a complicated role in tumor immune and inflammatory environments, which requires further research.

Gene methylation levels affect the immunogenicity of tumor cells and the tumor immune microenvironment. Using METHSRV and UALCAN, we analyzed the relationship between AQP5 methylation levels and PAAD clinical data. The results showed that hypomethylation of AQP5 was more common in patients with high-grade and advanced PAAD, suggesting that hypomethylation of AQP5 is more likely to promote tumorigenesis. We also found that CpG islands of AQP5 methylation were associated with unfavorable prognosis, suggesting that the AQP5 methylation level could be used as a prognostic marker of pancreatic cancer.

Our study has some limitations. Firstly, gene expression analysis based on an open-source database may not be sufficiently accurate. Further experiments are needed to explore the mechanisms of AQP5 in PAAD and its interactions with tumor immunity using *in vitro* and *in vivo* models. Second, how AQP5 expression and methylation affect immune cells and how its related genes promote the occurrence and development of PAAD in their molecular pathways are worthy of further study.

In conclusion, our data revealed that AQP5 can be used as a biomarker for PAAD prognosis. The expression and methylation status of AQP5 are not only related to immune cell infiltration but also related to immune modulators. This study is significant for the early diagnosis and treatment of PAAD, which also indicates the direction of further mechanistic research and lays a foundation for the design of targeted drugs for clinical transformation research in the future.

## Data Availability Statement

The datasets presented in this study can be found in online repositories. The names of the repository/repositories and accession number(s) can be found at: https://www.ncbi.nlm.nih.gov/geo/, GSE16515; https://www.ncbi.nlm.nih.gov/geo/, GSE71729.

## Ethics Statement

The studies involving human participants were reviewed and approved by the Ethics Committee of Drug Clinical Trials, Xijing Hospital, Air Force Medical University. The patients/participants provided their written informed consent to participate in this study.

## Author Contributions

ZL, GC, and DW designed the study. HS and ZY performed the experiment and data analysis. TD and YZ conducted the IHC and Western blot assay. KZ and DW contributed to manuscript writing, reviewing, and revision. All authors contributed to the article and approved the submitted version.

## Funding

This study was supported by the grants from the Key R&D Program of Ningxia Province (Project Number:4162021BEG03037) and the National Natural Science Foundation of China (Project Number:2017YFC1308603).

## Conflict of Interest

The authors declare that the research was conducted in the absence of any commercial or financial relationships that could be construed as a potential conflict of interest.

## Publisher’s Note

All claims expressed in this article are solely those of the authors and do not necessarily represent those of their affiliated organizations, or those of the publisher, the editors and the reviewers. Any product that may be evaluated in this article, or claim that may be made by its manufacturer, is not guaranteed or endorsed by the publisher.
